# A robust approach for computing solutions of fractional-order two-dimensional Helmholtz equation

**DOI:** 10.1038/s41598-024-54870-8

**Published:** 2024-02-20

**Authors:** Muhammad Nadeem, Zitian Li, Devendra Kumar, Yahya Alsayaad

**Affiliations:** 1 School of Mathematics and Statistics, Qujing Normal University, Qujing, 655011 China; 2 Department of Mathematics, University of Rajasthan, Jaipur, Rajasthan 302004 India; 3 Department of Physics, Hodeidah University, Al-Hudaydah, Yemen

**Keywords:** Elzaki transform, Fractional derivative, Helmholtz equation, Residual power series method, Analytical results, Applied mathematics, Software

## Abstract

The Helmholtz equation plays a crucial role in the study of wave propagation, underwater acoustics, and the behavior of waves in the ocean environment. The Helmholtz equation is also used to describe propagation through ocean waves, such as sound waves or electromagnetic waves. This paper presents the Elzaki transform residual power series method (${\mathbb {E}}$T-RPSM) for the analytical treatment of fractional-order Helmholtz equation. To develop this scheme, we combine Elzaki transform (${\mathbb {E}}$T) with residual power series method (RPSM). The fractional derivatives are described in Caputo sense. The ${\mathbb {E}}$T is capable of handling the fractional order and turning the problem into a recurrence form, which is the novelty of our paper. We implement RPSM in such a way that this recurrence relation generates the results in the form of an iterative series. Two numerical applications are considered to demonstrate the efficiency and authenticity of this scheme. The obtained series are determined very quickly and converge to the exact solution only after a few iterations. Graphical plots and absolute error are shown to observe the authenticity of this suggested approach.

## Introduction

The realm of fractional calculus (FC) has garnered profound interest owing to its diverse and impactful applications across scientific and engineering domains. FC has witnessed recent exploration in an array of physical phenomena, encompassing chemistry, physics, dynamic systems, engineering, and mathematical biology^1–4^. However, the computational intricacies entailed by fractional operators have added a layer of complexity to these investigations, making the analytical solution of fractional problems a formidable challenge.

Fortunately, the scientific community has responded with a wealth of innovative methodologies designed to conquer the challenges posed by fractional-order differential problems. These include the Homotopy perturbation scheme^5^, the Differential approach^6^, the Laplace homotopy strategy^7^, the Elzaki Adomian Decomposition Method^8^, the Modified extended tanh-function approach^9^, the Shehu transform^10^, the Variational iteration method^11^, the Jacobi collocation^12^, the Bivariate fractional power series^13^, the Legendre wavelet strategy^14^, the Natural decomposition scheme^15^, the Modified Kudryashov approach^16^, the Fractional reduced transform method^17^, the Residual power series method^18^, and the Chebyshev polynomial approach^19^. These methodologies collectively represent the scientific community’s dedication to advancing the field of fractional calculus, providing researchers with a rich toolkit for conquering complex problems in a myriad of disciplines.

Besides, the Helmholtz equation is a partial differential equation that appears in various areas of science and engineering, particularly in physics and wave-related phenomena. It is named after the German physicist Hermann von Helmholtz, who made significant contributions to the study of waves and their mathematical representation. In the Cartesian coordinate system, we consider a two-dimensional nonhomogeneous isotropic medium whose speed is *c*, then the wave solution is $\vartheta (\xi ,\eta )$, which corresponds to a harmonic source $\Phi (\xi ,\eta )$ oscillating at a constant frequency $\omega >0$ and satisfying the scalar Helmholtz equation on a specified area *R* such that,

1 $$\begin{aligned} \frac{\partial ^{\alpha }}{\partial \xi ^{\alpha }}\vartheta (\xi ,\eta )+\frac{\partial ^{2}}{\partial \eta ^{2}}\vartheta (\xi ,\eta )+\lambda \vartheta (\xi ,\eta )=-\Phi (\xi ,\eta ), \qquad {0<\alpha \le 1}{,} \end{aligned}$$

where $\vartheta (\xi ,\eta )$ is a sufficiently differentiable function on the boundary of *R*, and $\Phi (\xi ,\eta )$ is a given function, $\lambda > 0$ is a constant number, and $\sqrt{\lambda }=\omega /c$ is the wavenumber with wavelength $2\pi /\sqrt{\lambda }$. Equation (1) is commonly referred to as the reduction of wave equation, which emerges straight out of the waveform and captures the phenomenon of time-independent mechanical propagation within a spatial domain. Over the years, numerous researchers have diligently devised various techniques to derive analytical solutions for the classical Helmholtz equations.

Mehmet et al.^20^ demonstrated RPSM for the numerical solutions of the time-fractional Rosenau–Hyman equation and showed that the obtained results are in agreement with the exact solutions both numerically and graphically. Marwan et al.^21^ analyzed the approximate solutions of the generalized fractional Burgers–Huxley equation using the residual power series method. In^22^, authors harnessed an analytical approach to tackle the formidable Helmholtz equation. In a similar vein, Momani and Abuasad^23^ adeptly applied an iteration scheme to approximate solutions for the Helmholtz equation. Gupta et al. ^24^ skillfully employed the homotopy perturbation scheme, yielding analytical results. In^25^, authors navigated a scheme to derive the results, expanding the realm of solvable problems. These noteworthy contributions have enriched our understanding of this intricate field.

On the other hand, RPSM is an effective method for dealing with fractional integral and differential equations of fractional order. It hinges on the foundational premise that the solution to the problem can be expansively represented as a power series. RPSM stands out as an elegant, swift, and efficient approach for deducing the coefficients constituting the power series solution. Arqub, in a notable work^26^, harnessed RPSM’s capabilities to calculate the coefficients of power series solutions for fuzzy differential problems. The true advantage of RPSM lies in its ability to tackle problems without necessitating perturbation, linearization, or discretization. This method yields power series solutions, effectively addressing both linear and highly nonlinear equations, making it a versatile tool in the mathematical toolkit.

One distinctive feature of RPSM is its propensity to produce iterations in the form of power series. This property has been particularly beneficial, as evident in numerous examples from the past several years. Furthermore, RPSM not only provides a robust framework for ensuring the convergence of the series solution but also significantly reduces the associated residual error, thus improving the accuracy of the solution. In addition to its effectiveness, RPSM boasts the advantage of saving valuable computational time, as it circumvents the intricate intricacies of linear-to-nonlinear transitions. An aspect that is worth noting is that the success of RPSM relies on an initial approximation guess, a trait that streamlines its usability. Unlike certain methods that require transitioning from low-order to higher-order equations or from simple linearity to complex nonlinearity, RPSM offers an efficient and direct route to addressing fractional differential equations.

In recent work, the applicability and promise of RPSM has been further validated for various complex problems^27,28^. This method’s elegance lies in its ability to provide solutions efficiently, accurately, and without the need for complex transformations, making it a valuable tool for researchers and practitioners alike. In this paper, we embark on a comprehensive study of the approximate solutions to the time-fractional Helmholtz equation. Our methodology, known as the Elzaki Transform-Residual Power Series Method (${\mathbb {E}}$T-RPSM), is a fusion of the ${\mathbb {E}}$T and RPSM, which yields results in the form of a fractional power series. This innovative approach begins by employing the ${\mathbb {E}}$T to transform fractional problems into a recurrence relation. Subsequently, the inverse ${\mathbb {E}}$T is applied to generate an algebraic equation. Simultaneously, the RPSM is harnessed to produce results in the form of a power series, guiding us toward exact solutions. The structure of this paper unfolds as follows: In Sect. 2, we provide a concise overview of fractional calculus and the Elzaki transform. The algorithm of ${\mathbb {E}}$T-RPSM is expounded in Sect. 3. To validate the efficacy of our approach, we present a series of numerical applications of Helmholtz equations in Sects. 4 and 5, where we discuss the corresponding results. Finally, we conclude our study in Sect. 6, offering a succinct summary of our findings and their implications.

## Preliminaries concept of Elzaki transform

This section provides a thorough introduction to the Elzaki transform and the fundamental idea of the Caputo fractional.

### Definition 2.1

The fractional integral operator of Riemann–Liouville of order $\alpha > 0$ is defined as follows^29^

$$\begin{aligned} J^{\alpha }\vartheta (\eta )={\left\{ \begin{array}{ll} &{}\dfrac{1}{\Gamma (\alpha )}\int _{0}^{\eta }\dfrac{\vartheta (s)}{(\eta -s)^{1-s}}\ ds=\dfrac{1}{\Gamma (\alpha )}\eta ^{\alpha -1}*\vartheta (\eta ), \quad \alpha>0, \ \eta >0,\\ &{} \vartheta (\eta ), \alpha =0{,} \end{array}\right. } \end{aligned}$$

where $\eta ^{\alpha -1}*\vartheta (\eta )$ is the convolution product of $\eta ^{\alpha -1}$ and $\vartheta (\eta )$.

For the Riemann–Liouville fractional integral, we have

$$\begin{aligned}&J^{\alpha }\eta ^{\beta }=\frac{\Gamma (\beta +1)}{\Gamma (\beta +\alpha +1)}{\eta ^{\alpha +\beta }}{,}\\&J^{\alpha }(\lambda \vartheta (\eta )+\mu \zeta (\eta ))=\lambda J^{\alpha }\vartheta (\eta )+\mu J^{\alpha }\zeta (\eta ){,} \end{aligned}$$

where $\lambda$ and $\mu$ are real constants.

### Definition 2.2

Let $\vartheta (\xi , \eta ): [0, \infty )\rightarrow {\mathbb {R}}$ be a function. The definition of the Caputo fractional derivative is^29^

$$\begin{aligned} D^{\alpha }\vartheta (\xi ,\eta )=&\dfrac{1}{\Gamma (n-\alpha )}\int _{0}^{\eta }(\eta -s)^{n-\alpha -1}\frac{\partial ^{n}\vartheta (\xi ,s)}{\partial s^{n}}\ ds{,} \quad n-1<\alpha \le n,\ n\in {\mathbb {N}}{.} \end{aligned}$$

For the Caputo derivative, we have

$$\begin{aligned}&1.\quad D^{\alpha }J^{\alpha }\vartheta (\eta )=\vartheta (\eta ),\\&2.\quad J^{\alpha }D^{\alpha }\vartheta (\eta )=\vartheta (\eta )-\sum _{i=0}^{n}\vartheta ^{i}(0)\frac{\eta ^{i}}{i!},\\&3.\quad D^{\alpha }\eta ^{\beta }={\left\{ \begin{array}{ll} &{} \frac{\Gamma (\beta +1)}{\Gamma (\beta +1-\alpha )}\eta ^{\beta -\alpha } \beta \ge \alpha ,\\ &{} 0, \beta <\alpha \end{array}\right. }\\&4.\quad D^{\alpha } c=0,\\&5.\quad D^{\alpha }(\lambda \vartheta (\eta )+\mu \zeta (\eta ))=\lambda D^{\alpha }\vartheta (\eta )+\mu D^{\alpha }\zeta (\eta ){,} \end{aligned}$$

where $\lambda$, $\mu$ and *c* are real constants.

### Definition 2.3

A power series of the form^21^

$$\begin{aligned} \sum _{m=0}^{\infty }\vartheta _{m}(\xi )(\eta -\eta _{0})^{m \alpha }=\vartheta _{0}(\xi )+\vartheta _{1}(\xi )(\eta -\eta _{0})^{\alpha } +\vartheta _{2}(\xi )(\eta -\eta _{0})^{2\alpha }+\cdots , \qquad {\xi \in I, \quad \eta _{0}\le \eta <\eta _{0}+R,} \end{aligned}$$

is called multiple fractional power series about $\eta =\eta _{0}$, where $\eta$ is a variable and $\vartheta _{m}$’s are functions of $\xi$ called the coefficients of the series.

### Theorem 2.1

^30^ Suppose that $\vartheta (\xi , \eta )$ has a multiple fractional power series representation at $\eta =\eta _{0}$ of the form

2 $$\begin{aligned} \vartheta (\xi , \eta )=\sum _{m=0}^{\infty } \vartheta _m(\xi , \eta )=\sum _{m=0}^{\infty } \vartheta _m(\xi )(\eta -\eta _0)^{m \alpha }, \end{aligned}$$

$$0<n-1<\alpha \le n,\ x \in I,\ \eta _0 \le \eta <\eta _0+R$$.

If $D_\eta ^{m \alpha } \vartheta (\xi , \eta )$ are continuous on $I \times (\eta _0, \eta _0+{\mathbb {R}}), m=$ $0,1,2, \ldots$, then coefficients $\vartheta _m(\xi )$ of above equations are given as

$$\begin{aligned} \vartheta _m(\xi )=\frac{D_\eta ^{m \alpha } \vartheta (\xi , \eta _0)}{\Gamma (m \alpha +1)}, m=0,1,2, \ldots , \end{aligned}$$

where $D_\eta ^{m \alpha }=\dfrac{\partial ^{m \alpha }}{\partial \eta ^{m \alpha }}=\dfrac{\partial ^\alpha }{\partial \eta ^\alpha } \cdot \dfrac{\partial ^\alpha }{\partial \eta ^\alpha } \cdots \dfrac{\partial ^\alpha }{\partial \eta ^\alpha }(m- times)$, and $R=\min _{c \in I} R_c$, in which $R_c$ is the radius of convergence of the fractional power series $\sum _{m=0}^{\infty } f_m(c)(\eta -\eta _0)^{m \alpha }$. According to the convergence of the classic residual power series method, there is a real number $\lambda \in (0,1)$, such that $\Vert \vartheta _m(\xi , \eta )\Vert \le \lambda \Vert \vartheta _{m-1}(\xi , \eta )\Vert , \eta \in (\eta _0, \eta _0+R)$.

### Theorem 2.2

^30^ The convergence of the fractional power series can occur in only three ways $\sum _{m=0}^{\infty } \vartheta _m(\xi )(\eta -\eta _0)^{m \alpha }$ such as

1. This series tends to convergence when $\eta =\eta _{0}$, which means that there is no radius of convergence.
2. This series can converge when $\eta \ge \eta _{0}$, which means that the radius of convergence is $\infty$.
3. This series can converge when $\eta _{0}\le \eta <\eta _{0}+R$, exists for real number of some positive integers *R* and diverges in case of $\eta >\eta _{0}+R$. In this scenario, *R* shows the radius of convergence for fractional power series.

### Definition 2.4

The ${\mathbb {E}}$T is defined as^31^

3 $$\begin{aligned} {\mathbb {E}}[\vartheta (\eta )]=R(s)=s \int _{0}^{\infty } e^{-\dfrac{\eta }{s}} \vartheta (\eta ) d \eta , \end{aligned}$$

where *s* is transform function of $\eta$. If *R*(*s*) is the ${\mathbb {E}}$T of ${\mathbb {E}}[\vartheta (\eta )]$, then $\vartheta (\eta )= {\mathbb {E}}^{-1}[R(s)]$ is call the inverse ${\mathbb {E}}$T.

**Propositions:**

The differential properties of ${\mathbb {E}}$T are

4 $$\begin{aligned} \begin{aligned} {\mathbb {E}}[\eta ^{n}]&=n!s^{n+2},\\ {\mathbb {E}}[\vartheta '(\eta )]&=\frac{{\mathbb {E}}[\vartheta (\eta )]}{s}-s \vartheta (0),\\ {\mathbb {E}}[\vartheta ''(\eta )]&=\frac{{\mathbb {E}}[\vartheta (\eta )]}{s^{2}}-\vartheta (0)-s \vartheta '(0),\\ {\mathbb {E}}[\vartheta ^{n}(\eta )]&=\frac{{\mathbb {E}}[\vartheta (\eta )]}{s^{n}}-\sum _{k=0}^{n-1} s^{2-n+k} \vartheta ^k(0),\\ {\mathbb {E}}[\eta ^{\alpha }]&=\int _{0}^{\infty }e^{-s \eta }\eta ^{\alpha }\ d\eta =s^{\alpha +1}\Gamma (\alpha +1),\ {\mathbb {R}}(\alpha )>0. \end{aligned} \end{aligned}$$

### Definition 2.5

The ${\mathbb {E}}$T in the sense of Caputo fractional-order is defined as

5 $$\begin{aligned} {\mathbb {E}}[D^{\alpha }\vartheta (\eta )]&=s^{-\alpha } {\mathbb {E}}[\vartheta (\eta )]-\sum _{k=0}^{m-1} s^{2-\alpha +k} \vartheta ^k(0), \qquad m-1<\alpha <m{,} \end{aligned}$$

### Definition 2.6

Let us define the gamma function^32^

$$\begin{aligned} \Gamma (\alpha )=\int _{0}^{\infty }e^{-\eta }\eta ^{\alpha -1}\partial \eta , \quad \alpha >0{.} \end{aligned}$$

## Algorithm of ${\mathbb {E}}$T-RPSM

In this section, we present the idea of $\mathbb {{\mathbb {E}}}$T-RPSM step by step. This new approach is formulated on the combination of Elzaki transform with RPSM for the solution of two-dimensional Helmholtz equations with Caputo derivatives. Therefore, consider the differential equation of fractional order $\alpha$ towards the $\xi$-space such as

6 $$\begin{aligned} D^{\alpha }_{\eta }\vartheta (\xi ,\eta )=L \vartheta (\xi ,\eta )+N \vartheta (\xi ,\eta )+g(\xi ,\eta ){,} \end{aligned}$$

with initial condition

7 $$\begin{aligned} \vartheta (0,\eta )=f(\eta ){.} \end{aligned}$$

*Step 1* Employing $\mathbb {{\mathbb {E}}}$T on Eq. (6), we get

$$\begin{aligned} {\mathbb {E}}\Big [D^{\alpha }_{\eta }\vartheta (\xi ,\eta )\Big ]={\mathbb {E}}\Big [L \vartheta (\xi ,\eta )+N \vartheta (\xi ,\eta )+g(\xi ,\eta )\Big ]{.} \end{aligned}$$

Applying the differentiation property of $\mathbb {{\mathbb {E}}}$T, we get

$$\begin{aligned} \frac{1}{s^{\alpha }}{\mathbb {E}}[\vartheta (\xi ,\eta )]-s^{2-\alpha }\vartheta (0,\eta )&={\mathbb {E}}\Big [L \vartheta (\xi ,\eta )+N \vartheta (\xi ,\eta )+g(\xi ,\eta )\Big ]{.} \end{aligned}$$

After solving and using the initial condition (8), we get

8 $$\begin{aligned} {\mathbb {E}}[\vartheta (\xi ,\eta )]=s^{2}f(\eta )+s^{\alpha }{\mathbb {E}}[g(\xi ,\eta )]+s^{\alpha }{\mathbb {E}}\Big [L \vartheta (\xi ,\eta )+N \vartheta (\xi ,\eta )\Big ]{.} \end{aligned}$$

*Step 2* The inverse $\mathbb {{\mathbb {E}}}$T on Eq. (8) yields as

9 $$\begin{aligned} \vartheta (\xi ,\eta )=F(\xi ,\eta )+{\mathbb {E}}^{-1}\Big [s^{\alpha }\Big \{L \vartheta (\xi ,\eta )+N \vartheta (\xi ,\eta )\Big \}\Big ]{,} \end{aligned}$$

where

$$\begin{aligned} F(\xi ,\eta )={\mathbb {E}}^{-1}\Big [s^{2}f(\eta )+s^{\alpha }{\mathbb {E}}[g(\xi ,\eta )]\Big ]{.} \end{aligned}$$

*Step 3* In case of using RPSM, we can express the results of Eq. (6) as an expanding of fractional power series at point $\xi =0$ such as

10 $$\begin{aligned} \vartheta (\xi , \eta )=\sum _{n=0}^{\infty }\vartheta _{n}(\eta )\frac{\xi ^{n\alpha }}{\Gamma (n\alpha +1)}. \end{aligned}$$

Now, we may define the truncated series $\vartheta _{k}(\xi , \eta )$ such as

11 $$\begin{aligned} \vartheta _{k}(\xi , \eta )=\vartheta _{0}+\sum _{n=1}^{k}\vartheta _{n}(\eta )\frac{\xi ^{k\alpha }}{\Gamma (k\alpha +1)}. \end{aligned}$$

where *k*th iterations of RPSM are derived by considering $k=1,2,3,\ldots$.

*Step 4* Consider that the residual function of Eq. (9) such as

$$\begin{aligned} {\text {Res}}\vartheta (\xi , \eta )=\vartheta (\xi ,\eta )-F(\xi ,\eta )-{\mathbb {E}}^{-1}\Big [s^{\alpha }\Big \{L \vartheta (\xi ,\eta )+N \vartheta (\xi ,\eta )\Big \}\Big ]{.} \end{aligned}$$

Thus, the *k*th residual function yields as

12 $$\begin{aligned} {\text {Res}}\vartheta _{k}(\xi , \eta )=\vartheta _{k}(\xi ,\eta )-F(\xi ,\eta )-{\mathbb {E}}^{-1}\Big [s^{\alpha }\Big \{L \vartheta _{k}(\xi ,\eta )+N \vartheta _{k}(\xi ,\eta )\Big \}\Big ]{.} \end{aligned}$$

The RPSM have the following facts such that

- $$\quad \displaystyle \lim _{k \rightarrow \infty } {\text {Res}}\vartheta _{k}(\xi , \eta )={\text {Res}}\vartheta (\xi , \eta )=0$$,
- $$\quad D^{m}_{\eta }{\text {Res}}\vartheta _{k}(\xi , 0)=0, \qquad m=0,1,2,\ldots$$.

*Step 5* Use this resulting series of (12) to the residual function of (11).

*Step 6* The terms of $\vartheta _n(\xi , \eta )$, for $n=1,2,3, \ldots$, can be derived by using the system of $D^{m}_{\eta } {\text {Res}}\vartheta _{n}(\xi , \eta )=0$ that provides the results in a form of fractional iterative series.

*Step 7* Using Eq. (12), we can derive the fractional iterative series that converges to the exact solution at $\alpha =2$.

## Numerical tests

This section discusses the use of ${\mathbb {E}}$T-RPSM for analytically solving Helmholtz equations with Caputo fractional orders. We examine two numerical applications to verify the legitimacy of ${\mathbb {E}}$T-RPSM and find that the results are in a fractional power series. The readers can examine the accuracy of ${\mathbb {E}}$T-RPSM through graphical displays whereas a comparison between the analytical and exact findings is demonstrated by the computation of absolute error.

### Example 1

Let us assume the time-fractional Helmholtz problem towards $\xi$-space such as

13 $$\begin{aligned} \frac{\partial ^{\alpha }\vartheta }{\partial \xi ^{\alpha }}+\frac{\partial ^{2}\vartheta }{\partial \eta ^{2}}-\vartheta =0, \qquad \qquad 1<\alpha \le 2, \end{aligned}$$

subjected to

14 $$\begin{aligned} \vartheta (0,\eta )=\eta , \qquad \vartheta _{\xi }(0,\eta )=0. \end{aligned}$$

Using the $\mathbb {{\mathbb {E}}}$T on Eq. (13) and solving it, we obtain

$$\begin{aligned} {\mathbb {E}}[\vartheta (\xi ,\eta )]=s^{2}\vartheta (0,\eta )-s^{\alpha }{\mathbb {E}} \Big [\frac{\partial ^{2}\vartheta }{\partial \eta ^{2}}-\vartheta \Big ]{.} \end{aligned}$$

The inverse $\mathbb {{\mathbb {E}}}$T yields as

$$\begin{aligned} \vartheta (\xi ,\eta )=\vartheta _{0}-{\mathbb {E}}^{-1}\Big [s^{\alpha }{\mathbb {E}} \Big (\frac{\partial ^{2}\vartheta }{\partial \eta ^{2}}-\vartheta \Big )\Big ]{.} \end{aligned}$$

Therefore, we can obtain the *k*th residual function such as

15 $$\begin{aligned} {\text {Res}}\vartheta _{k}(\xi ,\eta )=\vartheta _{k}(\xi ,\eta )-\vartheta _{0} +{\mathbb {E}}^{-1}\Big [s^{\alpha }{\mathbb {E}}\Big (\frac{\partial ^{2}\vartheta _{k}}{\partial \eta ^{2}}-\vartheta _{k}\Big )\Big ]{.} \end{aligned}$$

Let’s us consider the solution of Eq. (13) is as follows

16 $$\begin{aligned} \vartheta (\xi , \eta )=\sum _{n=0}^{\infty }\vartheta _{n}(\eta )\frac{\xi ^{n\alpha }}{\Gamma (n\alpha +1)}. \end{aligned}$$

Let $\vartheta _{k}(\xi , \eta )$ shows the *k*th transform function as

17 $$\begin{aligned} \vartheta _{k}(\xi , \eta )=\vartheta _{0} +\sum _{n=1}^{k}\vartheta _{n}(\eta )\frac{\xi ^{k\alpha }}{\Gamma (k\alpha +1)}. \end{aligned}$$

Let $k=1, 2, 3, 4$, the following iterations are obtained from Eq. (17)

18 $$\begin{aligned} \begin{aligned} \vartheta _{1}(\xi , \eta )&=\vartheta _{0}+\vartheta _{1}\frac{\xi ^{\alpha }}{\Gamma (\alpha +1)}{,}\\ \vartheta _{2}(\xi , \eta )&=\vartheta _{0}+\vartheta _{1}\frac{\xi ^{\alpha }}{\Gamma (\alpha +1)} +\vartheta _{2}\frac{\xi ^{2\alpha }}{\Gamma (2\alpha +1)}{,}\\ \vartheta _{3}(\xi ,\eta )&=\vartheta _{0}+\vartheta _{1}\frac{\xi ^{\alpha }}{\Gamma (\alpha +1)} +\vartheta _{2}\frac{\xi ^{2\alpha }}{\Gamma (2\alpha +1)}+\vartheta _{3}\frac{\xi ^{3\alpha }}{\Gamma (3\alpha +1)}{,}\\ \vartheta _{4}(\xi ,\eta )&=\vartheta _{0}+\vartheta _{1}\frac{\xi ^{\alpha }}{\Gamma (\alpha +1)} +\vartheta _{2}\frac{\xi ^{2\alpha }}{\Gamma (2\alpha +1)}+\vartheta _{3} \frac{\xi ^{3\alpha }}{\Gamma (3\alpha +1)}+\vartheta _{4}\frac{\xi ^{4\alpha }}{\Gamma (4\alpha +1)}{.} \end{aligned} \end{aligned}$$

Now using the system of Eq. (18) into (16) and using ${\text {Res}}\vartheta _{1}(\xi ,\eta )\rightarrow 0$, ${\text {Res}}\vartheta _{2}(\xi ,\eta )\rightarrow 0$, ${\text {Res}}\vartheta _{3}(\xi ,\eta )\rightarrow 0$, ${\text {Res}}\vartheta _{4}(\xi ,\eta )\rightarrow 0$, we can obtain the following iteration results. Thus, the following iteration can be obtained as follows

$$\begin{aligned} \vartheta _{1}(\eta )&=\eta {,}\\ \vartheta _{2}(\eta )&=\eta {,}\\ \vartheta _{3}(\eta )&=\eta {,}\\ \vartheta _{4}(\eta )&=\eta {,} \end{aligned}$$

continuing these iterations, the *n*th residual function of Eq. (17) yields as

19 $$\begin{aligned} \begin{aligned} \vartheta (\xi , \eta )=&\,\vartheta _{0}(\eta )+\vartheta _{1}(\eta )\frac{\xi ^{\alpha }}{\Gamma (\alpha +1)} +\vartheta _{2}(\eta )\frac{\xi ^{2\alpha }}{\Gamma (2\alpha +1)}+\vartheta _{3}(\eta ) \frac{\xi ^{3\alpha }}{\Gamma (3\alpha +1)}+\vartheta _{4}(\eta )\frac{\xi ^{4\alpha }}{\Gamma (4\alpha +1)}+\cdots \\ =&\,\eta \Big (1+\frac{\xi ^{\alpha }}{\Gamma (\alpha +1)}+\frac{\xi ^{2\alpha }}{\Gamma (2\alpha +1)} +\frac{\xi ^{3\alpha }}{\Gamma (3\alpha +1)}+\frac{\xi ^{4\alpha }}{\Gamma (4\alpha +1)}+\cdots \Big ). \end{aligned} \end{aligned}$$

we can also write it as follows,

20 $$\begin{aligned} \vartheta (\xi , \eta )=\eta \sum _{k=0}^{\infty }\frac{\xi ^{k\alpha }}{\Gamma (1+k\alpha )}{.} \end{aligned}$$

The exact results yield as follows depending on the Mittag–Leffler function’s property

21 $$\begin{aligned} \vartheta (\xi , \eta )=\eta E_{\alpha }(\xi ^{\alpha }){,} \end{aligned}$$

where $1<\alpha \le 2$ and $E_{\alpha }(z)$ is represented by Mittag–Leffler function which turns as for $\alpha =2$

$$\begin{aligned} \vartheta (\xi , \eta )=\sum _{k=0}^{\infty }\frac{\xi ^{2k}}{\Gamma (1+2k)}=\sum _{k=0}^{\infty }\frac{\xi ^{2k}}{(2k)!}=\cosh \xi {.} \end{aligned}$$

Hence, the precise solution for Eq. (13) in $\xi$-space at $\alpha$ = 2 is

22 $$\begin{aligned} \vartheta (\xi , \eta )=\eta \cosh \xi {.} \end{aligned}$$

**Table 1:** Absolute error among the ${\mathbb {E}}$T-RPSM results and the precise results of $\vartheta (\xi , \eta )$ along $\xi$-space at different order of $\alpha$ for Example 1.

$(\xi ,\eta )$	${\mathbb {E}}$T-RPSM	${\mathbb {E}}$T-RPSM	${\mathbb {E}}$T-RPSM	Exact results	Absolute error
	$\alpha =1$	$\alpha =1.5$	$\alpha =2$		
(0.2, 0.2)	0.24426	0.21372	0.20401	0.20401	00000
(0.4, 0.4)	0.59626	0.48051	0.43242	0.43242	00000
(0.6, 0.6)	1.08960	0.83252	0.71127	0.71127	00000
(0.8, 0.8)	1.07642	1.30448	1.06994	1.06995	0.00001
(1.0, 1.0)	2.66667	1.93802	1.54306	1.54308	0.00002
(1.2, 1.2)	3.84960	2.78431	2.17266	2.17279	0.00013
(1.4, 1.4)	5.37227	3.90639	3.01073	3.01126	0.00053
(1.6, 1.6)	7.30027	5.38159	4.12219	4.12394	0.00175
(1.8, 1.8)	8.21280	6.05429	5.58835	5.59345	0.00510
(2.0, 2.0)	12.66670	9.78664	7.51111	7.52439	0.01328

**Figure 1:**
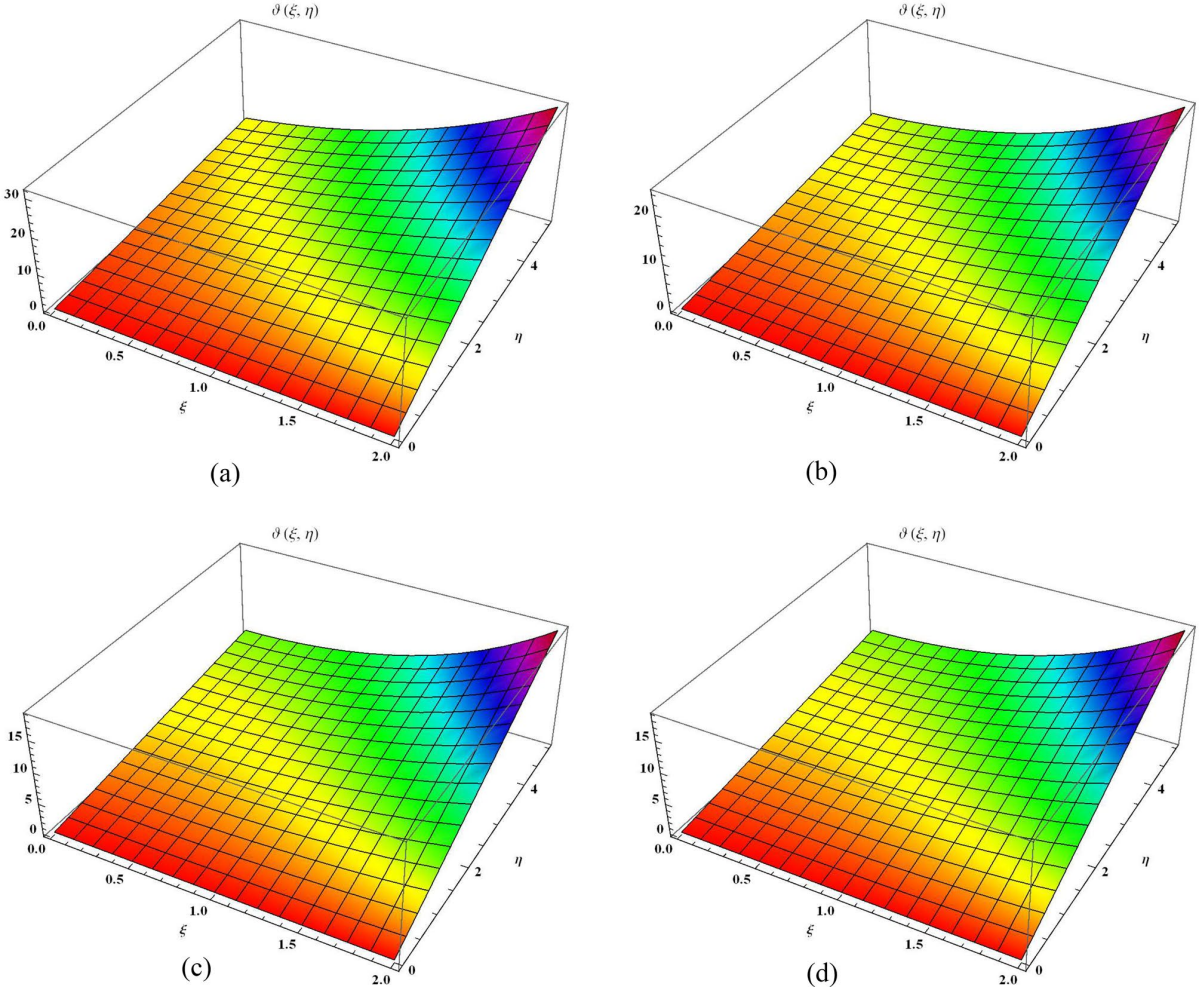
The 3D comparison of ${\mathbb {E}}$T-RPSM solution of $\vartheta (\xi , \eta )$ along $\xi$-space at various fractional order with the exact solution. (**a**) 3D $\mathbb{E}$T-RPSM solution of $\vartheta(\xi, \eta)$ along $\xi$-space at $\alpha=1$. (**b**) 3D$\mathbb{E}$T-RPSM solution of $\vartheta(\xi, \eta)$ along $\xi$-space at $\alpha=1.5$. (**c**) 3D $\mathbb{E}$T-RPSM solution of $\vartheta(\xi, \eta)$ along $\xi$-space at $\alpha=2$. (**d**) 3D exact solution of $\vartheta(\xi, \eta)$ along $\xi$-space

**Figure 2:**
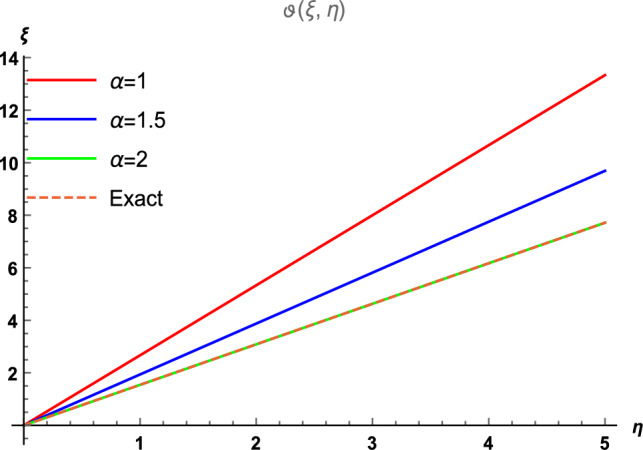
The 2D comparison of $\vartheta (\xi , \eta )$ along $\xi$-space at different fractional order with the precise results.

Similarly, the time-fractional Helmholtz problem towards $\eta$-space such as

23 $$\begin{aligned} \frac{\partial ^{\alpha }\vartheta }{\partial \eta ^{\alpha }}+\frac{\partial ^{2}\vartheta }{\partial \xi ^{2}}-\vartheta =0, \qquad \qquad 1<\alpha \le 2{,} \end{aligned}$$

subjected to

24 $$\begin{aligned} \vartheta (\xi ,0)=\xi {.} \end{aligned}$$

Using ${\mathbb {E}}$T-RPSM, we can obtain the series for Eq. (23) such as

$$\begin{aligned} \vartheta (\xi ,\eta )=&\vartheta _{0}(\xi )+\vartheta _{1}(\xi )\frac{\eta ^{\alpha }}{\Gamma (\alpha +1)} +\vartheta _{2}(\xi )\frac{\eta ^{2\alpha }}{\Gamma (2\alpha +1)}+\vartheta _{3}(\xi ) \frac{\eta ^{3\alpha }}{\Gamma (3\alpha +1)}+\vartheta _{4}(\xi )\frac{\eta ^{4\alpha }}{\Gamma (4\alpha +1)}+\cdots \\ =&\xi \Big (1+\frac{\eta ^{\alpha }}{\Gamma (\alpha +1)}+\frac{\eta ^{2\alpha }}{\Gamma (2\alpha +1)} +\frac{\eta ^{3\alpha }}{\Gamma (3\alpha +1)}+\frac{\eta ^{4\alpha }}{\Gamma (4\alpha +1)}+\cdots \Big ). \end{aligned}$$

We can also write it as follows

$$\begin{aligned} \vartheta (\xi , \eta )=\xi \sum _{k=0}^{\infty }\frac{\eta ^{k\alpha }}{\Gamma (1+k\alpha )}{.} \end{aligned}$$

The Mittag–Leffler function’s property turns the precise solution for Eq. (23) in $\eta$-space at $\alpha =2$ is

25 $$\begin{aligned} \vartheta (\xi , \eta )=\xi \cosh \eta {.} \end{aligned}$$

### Example 2

Next, again assume the time-fractional Helmholtz problem towards $\xi$-space such as

26 $$\begin{aligned} \frac{\partial ^{\alpha }\vartheta }{\partial \xi ^{\alpha }}+\frac{\partial ^{2}\vartheta }{\partial \eta ^{2}}+5\vartheta =0, \qquad \qquad 1<\alpha \le 2{,} \end{aligned}$$

subjected to

27 $$\begin{aligned} \vartheta (0,\eta )=\eta , \qquad \vartheta _{\xi }(0,\eta )=0. \end{aligned}$$

Using the $\mathbb {{\mathbb {E}}}$T on Eq. (26) and solving it, we obtain

28 $$\begin{aligned} {\mathbb {E}}[\vartheta (\xi ,\eta )]=s^{2}\vartheta (0,\eta )-s^{\alpha }{\mathbb {E}} \Big [\frac{\partial ^{2}\vartheta }{\partial \eta ^{2}}+5\vartheta \Big ]{.} \end{aligned}$$

The inverse $\mathbb {{\mathbb {E}}}$T yields as

29 $$\begin{aligned} \vartheta (\xi ,\eta )=\vartheta _{0}-{\mathbb {E}}^{-1}\Big [s^{\alpha }{\mathbb {E}} \Big (\frac{\partial ^{2}\vartheta }{\partial \eta ^{2}}+5\vartheta \Big )\Big ]{.} \end{aligned}$$

Therefore, we can obtain the *k*th residual as

30 $$\begin{aligned} {\text {Res}}\vartheta _{k}(\xi ,\eta )=\vartheta _{k}(\xi ,\eta )-\vartheta _{0} +{\mathbb {E}}^{-1}\Big [s^{\alpha }{\mathbb {E}}\Big (\frac{\partial ^{2}\vartheta _{k}}{\partial \eta ^{2}}+5\vartheta _{k}\Big )\Big ]{.} \end{aligned}$$

Let’s us consider the solution of Eq. (26) is as follows

31 $$\begin{aligned} \vartheta (\xi , \eta )=\sum _{n=0}^{\infty }\vartheta _{n}(\eta )\frac{\xi ^{n\alpha }}{\Gamma (n\alpha +1)}. \end{aligned}$$

Let $\vartheta _{k}(\xi , \eta )$denotes the *k*th transform function as

32 $$\begin{aligned} \vartheta _{k}(\xi , \eta )=\vartheta _{0}+\sum _{n=1}^{k}\vartheta _{n}(\eta )\frac{\xi ^{k\alpha }}{\Gamma (k\alpha +1)}. \end{aligned}$$

Let $k=1, 2, 3, 4$, the following iterations are obtained from Eq. (32)

33 $$\begin{aligned} \begin{aligned} \vartheta _{1}(\xi , \eta )&=\vartheta _{0}+\vartheta _{1}\frac{\xi ^{\alpha }}{\Gamma (\alpha +1)}{,}\\ \vartheta _{2}(\xi , \eta )&=\vartheta _{0}+\vartheta _{1}\frac{\xi ^{\alpha }}{\Gamma (\alpha +1)} +\vartheta _{2}\frac{\xi ^{2\alpha }}{\Gamma (2\alpha +1)}{,}\\ \vartheta _{3}(\xi ,\eta )&=\vartheta _{0}+\vartheta _{1}\frac{\xi ^{\alpha }}{\Gamma (\alpha +1)} +\vartheta _{2}\frac{\xi ^{2\alpha }}{\Gamma (2\alpha +1)}+\vartheta _{3}\frac{\xi ^{3\alpha }}{\Gamma (3\alpha +1)}{,}\\ \vartheta _{4}(\xi ,\eta )&=\vartheta _{0}+\vartheta _{1}\frac{\xi ^{\alpha }}{\Gamma (\alpha +1)} +\vartheta _{2}\frac{\xi ^{2\alpha }}{\Gamma (2\alpha +1)}+\vartheta _{3} \frac{\xi ^{3\alpha }}{\Gamma (3\alpha +1)}+\vartheta _{4}\frac{\xi ^{4\alpha }}{\Gamma (4\alpha +1)}{.} \end{aligned} \end{aligned}$$

Now using the system of equation (33) into (31) and using ${\text {Res}}\vartheta _{1}(\xi ,\eta )\rightarrow 0$, ${\text {Res}}\vartheta _{2}(\xi ,\eta )\rightarrow 0$, ${\text {Res}}\vartheta _{3}(\xi ,\eta )\rightarrow 0$, ${\text {Res}}\vartheta _{4}(\xi ,\eta )\rightarrow 0$, we can obtain the following iteration results. Thus, the following iteration can be obtained as follows

$$\begin{aligned} \vartheta _{1}(\eta )&=-5\eta {,}\\ \vartheta _{2}(\eta )&=-25\eta {,}\\ \vartheta _{3}(\eta )&=-125\eta {,}\\ \vartheta _{4}(\eta )&=-625\eta {,} \end{aligned}$$

continuing these iterations, the *n*th residual function of Eq. (32) yields as

34 $$\begin{aligned} \begin{aligned} \vartheta (\xi , \eta )=&\,\vartheta _{0}(\eta )+\vartheta _{1}(\eta )\frac{\xi ^{\alpha }}{\Gamma (\alpha +1)} +\vartheta _{2}(\eta )\frac{\xi ^{2\alpha }}{\Gamma (2\alpha +1)}+\vartheta _{3}(\eta )\frac{\xi ^{3\alpha }}{\Gamma (3\alpha +1)}+\vartheta _{4}(\eta )\frac{\xi ^{4\alpha }}{\Gamma (4\alpha +1)}+\cdots \\ =&\,\eta \Big (1-5\frac{\xi ^{\alpha }}{\Gamma (\alpha +1)}-25\frac{\xi ^{2\alpha }}{\Gamma (2\alpha +1)} -125\frac{\xi ^{3\alpha }}{\Gamma (3\alpha +1)}-625\frac{\xi ^{4\alpha }}{\Gamma (4\alpha +1)}+\cdots \Big ). \end{aligned} \end{aligned}$$

we can also write it as follows

35 $$\begin{aligned} \vartheta (\xi , \eta )=\eta \sum _{k=0}^{\infty }\frac{(-5\xi ^{\alpha })^{k}}{\Gamma (1+k\alpha )}{.} \end{aligned}$$

The exact results yield as follows depending on the Mittag–Leffler function’s property

$$\begin{aligned} \vartheta (\xi , \eta )=\eta E_{\alpha }(-5\xi ^{\alpha }){,} \end{aligned}$$

where $1<\alpha \le 2$ and $E_{\alpha }(z)$ is represented by Mittag–Leffler function which turns as for $\alpha =2$

36 $$\begin{aligned} E_{2}(-5\xi ^{2})=\sum _{k=0}^{\infty }\frac{(-5\xi ^{2})^{k}}{\Gamma (1+2k)} =\sum _{k=0}^{\infty }\frac{(-1)^{k}(\sqrt{5}\xi )^{2k}}{(2k)!}{.} \end{aligned}$$

Hence, the precise solution for Eq. (26) in $\xi$-space at $\alpha$ = 2 is

37 $$\begin{aligned} \vartheta (\xi , \eta )=\eta \cos \sqrt{5} \xi {.} \end{aligned}$$

**Table 2:** Absolute error among the ${\mathbb {E}}$T-RPSM results and the precise results of $\vartheta (\xi , \eta )$ along $\xi$-space at different order of $\alpha$ for Example 2.

$(\xi ,\eta )$	${\mathbb {E}}$T-RPSM	${\mathbb {E}}$T-RPSM	${\mathbb {E}}$T-RPSM	Exact results	Absolute error
	$\alpha =1$	$\alpha =1.5$	$\alpha =2$		
(0.1, 0.1)	0.06067	0.08851	0.09751	0.09751	00000
(0.2, 0.2)	0.75000	0.13905	0.18033	0.18033	00000
(0.3, 0.3)	0.08203	0.14535	0.23499	0.23499	00000
(0.4, 0.4)	0.13333	0.11201	0.25038	0.25038	00000
(0.5, 0.5)	0.32421	0.04952	0.21872	0.21872	00000
(0.6, 0.6)	0.82500	− 0.02838	0.13629	0.13629	00000
(0.7, 0.7)	1.91224	− 0.10588	0.00390	0.00388	0.00002
(0.8, 0.8)	4.00000	− 0.16428	− 0.17299	− 0.17306	0.00007
(0.9, 0.9)	7.67109	− 0.17913	− 0.38443	− 0.38470	0.00027
(1.0, 1.0)	13.70830	− 0.11464	− 0.61644	− 0.61727	0.00083

**Figure 3:**
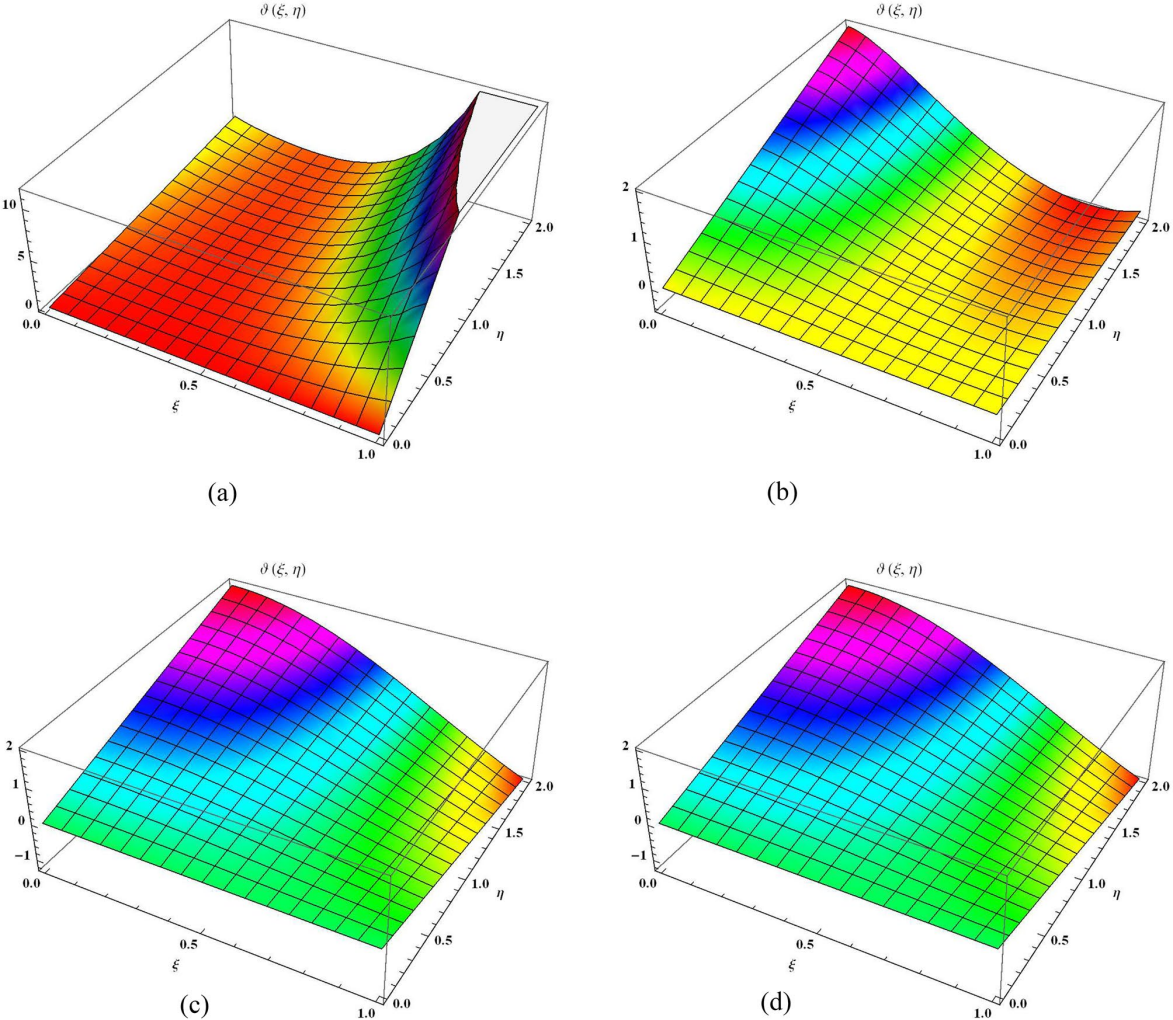
The 3D comparison of ${\mathbb {E}}$T-RPSM solution of $\vartheta (\xi , \eta )$ along $\xi$-space at various fractional order with the exact solution. (**a**) 3D $\mathbb{E}$T-RPSM solution of $\vartheta(\xi, \eta)$ along $\xi$-space at $\alpha=1$. (**b**) 3D $\mathbb{E}$T-RPSM solution of $\vartheta(\xi, \eta)$ along $\xi$-space at $\alpha=1.5$. (**c**) 3D $\mathbb{E}$T-RPSM solution of $\vartheta(\xi, \eta)$ along $\xi$-space at $\alpha=2$. (**d**) 3D exact solution of $\vartheta(\xi, \eta)$ along $\xi$-space

**Figure 4:**
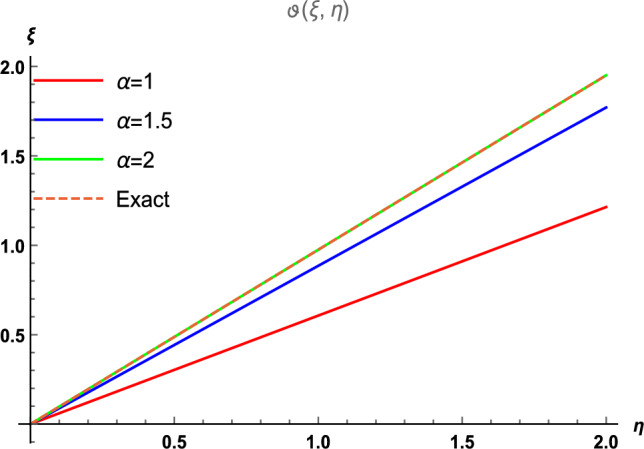
The 2D comparison of $\vartheta (\xi , \eta )$ along $\xi$-space at different fractional order with the precise results.

Similarly, the time-fractional Helmholtz problem towards $\eta$-space such as

38 $$\begin{aligned} \frac{\partial ^{\alpha }\vartheta }{\partial \eta ^{\alpha }}+\frac{\partial ^{2}\vartheta }{\partial \xi ^{2}}-\vartheta =0, \qquad \qquad 1<\alpha \le 2{,} \end{aligned}$$

subjected to

39 $$\begin{aligned} \vartheta (\xi ,0)=\xi {.} \end{aligned}$$

Using ${\mathbb {E}}$T-RPSM, we can obtain the series for Eq. (38) such as

$$\begin{aligned} \vartheta (\xi , \eta )=&\,\vartheta _{0}(\xi )+\vartheta _{1}(\xi )\frac{\eta ^{\alpha }}{\Gamma (\alpha +1)} +\vartheta _{2}(\xi )\frac{\eta ^{2\alpha }}{\Gamma (2\alpha +1)}+\vartheta _{3}(\xi )\frac{\eta ^{3\alpha }}{\Gamma (3\alpha +1)}+\vartheta _{4}(\xi )\frac{\eta ^{4\alpha }}{\Gamma (4\alpha +1)}+\cdots \\ =&\,\xi \Big (1-5\frac{\eta ^{\alpha }}{\Gamma (\alpha +1)}-25\frac{\eta ^{2\alpha }}{\Gamma (2\alpha +1)} -125\frac{\eta ^{3\alpha }}{\Gamma (3\alpha +1)}-625\frac{\eta ^{4\alpha }}{\Gamma (4\alpha +1)}+\cdots \Big ). \end{aligned}$$

we can also write it as follows

40 $$\begin{aligned} \vartheta (\xi , \eta )=\xi \sum _{k=0}^{\infty }\frac{(-5\eta ^{\alpha })^{k}}{\Gamma (1+k\alpha )}{.} \end{aligned}$$

The Mittag–Leffler function’s property turns the precise solution for Eq. (38) in $\eta$-space at $\alpha =2$ is

41 $$\begin{aligned} \vartheta (\xi , \eta )=\xi \cos \sqrt{5} \eta {.} \end{aligned}$$

## Results and discussion

This part provides a comprehensive explanation of the graphical demonstrations for 2D Helmholtz equation involving Caputo order. In Fig. 1, we meticulously partitioned in four distinct sectors, each corresponding to a different fractional order, while considering the parameter ranges of $0\le \xi \le 2$ and $0\le \eta \le 5$. Figure 1a, b display the outcomes derived from the outcomes of ${\mathbb {E}}$T-RPSM at $\alpha =1$ and $\alpha =1.5$ respectively. Figure 1c showcases results attained at $\alpha =2$ using the ${\mathbb {E}}$T-RPSM method, and Fig. 1d presents the visuals of the precise results. Additionally, Table 1 furnishes the absolute error values among the ${\mathbb {E}}$T-RPSM and the precis results for fractional orders $\alpha =1, 1.5, 2$. Notably, the results derived by the ${\mathbb {E}}$T-RPSM method at $\alpha =2$ exhibit a remarkable alignment with the exact solution. Furthermore, it is worth noting that the absolute error decreases as the values of $\xi$ and $\eta$ decrease. Moving forward, Fig. 2 provides a comprehensive overview of 2D graphical representation of $\vartheta (\xi ,\eta )$ across the $\xi$-space for various fractional orders. This illustration is performed for the parameter range of $0 \le \eta \le 5$, with $\xi =1$, and is subsequently compared with the exact graphical structure.

Similarly, Fig. 3 is divided into four distinct sections, each corresponding to different fractional orders, while considering the parameter ranges of $0\le \xi \le 1$ and $0\le \eta \le 2$. Figure 3a, b represent the results derived through the ${\mathbb {E}}$T-RPSM method at $\alpha =1$ and $\alpha =1.5,$ respectively. Figure 3c showcases results attained at a fractional order of 2 using the ${\mathbb {E}}$T-RPSM method, while Fig. 3d presents the visuals of the precis results. Once again, a strong alignment is observed between the graphical visuals of the ${\mathbb {E}}$T-RPSM method and the precis results, confirming the reliability of our approach. Table 2 provides the absolute error values among the ${\mathbb {E}}$T-RPSM and the precis results for fractional orders $\alpha =1, 1.5, 2.$ Similar to previous case, the results derived at a fractional order of $\alpha =2$ exhibit a high degree of agreement with the precis results, and the absolute error minimizes as the values of $\xi$ and $\eta$ decrease. Finally, Fig. 4 offers a 2D graphical representation of $\vartheta (\xi ,\eta )$ along the $\xi$-space for various fractional orders. This presentation is conducted for the parameter range of $0 \le \eta \le 2,$ with $\xi =0.1,$ and is subsequently compared with the exact graphical structure. The overlapping alignment between the two sets of results serves as compelling evidence of the validity and authenticity of our proposed scheme for solving the fractional Helmholtz problems.

## Conclusion

In this paper, we have successfully harnessed the power of ${\mathbb {E}}$T-RPSM method to obtain analytical solutions for fractional-order Helmholtz equations. The complete procedure of determining the analytical solutions for fractional-order Helmholtz equations is explained very beautifully step by step with two numerical problems. The comparison of the analytical solutions and the exact solution of the time-fractional Helmholtz equations with different fractional order $\alpha$ are presented through tabular form. We created the iteration series for both examples to show that as the parameter of convergence increased, the absolute error reduced. Our approach yields results in the form of rapid-converging series, eliminating the need for variable restrictions and hypotheses. To demonstrate the efficiency and accuracy of ${\mathbb {E}}$T-RPSM-generated analytical solutions, we present absolute error analysis, showcasing their proximity to the exact solution. Furthermore, we provide 2D and 3D graphical representations that offer insightful visual interpretations across various fractional orders. Our work underscores the effectiveness and swiftness of the proposed method in producing iterative series. Looking ahead, we intend to extend the applicability of this approach to other fractional problem involving nonlinear cases with fractal-fractional order equations and explore its utility in addressing various nonlinear challenges within the realms of science and engineering.

## Data Availability

All data generated or analysed during this study are included in this published article.
